# Understanding Cyclic Fatigue in Three Nickel–Titanium Pediatric Files: An In Vitro Study for Enhanced Patient Care

**DOI:** 10.3390/medicina61050830

**Published:** 2025-04-30

**Authors:** Alwaleed Abushanan, Rajashekhara Bhari Sharanesha, Fahd Aljarbou, Hadi Alamri, Mohammed Hamad Almasud, Abdulfatah AlAzmah, Sara Alghamdi, Mubashir Baig Mirza

**Affiliations:** 1 Department of Pediatric Dentistry, College of Dentistry, Prince Sattam bin Abdulaziz University, Al-Kharj 11942, Saudi Arabia; a.alazmah@psau.edu.sa (A.A.); sar.alghamdi@psau.edu.sa (S.A.); 2 Restorative Dental Science Department, College of Dentistry, King Saud University, Riyadh 11545, Saudi Arabia; faljarbou@ksu.edu.sa; 3 Department of Dentistry, King Faisal Specialist Hospital and Research Center, Riyadh 11211, Saudi Arabia; halamri4@kfshrc.edu.sa; 4 College of Dentistry, King Saud University, Riyadh 11545, Saudi Arabia; rajupedo@gmail.com; 5 Conservative Dental Sciences Department, College of Dentistry, Prince Sattam bin Abdulaziz University, Al-Kharj 11942, Saudi Arabia; m.mirza@psau.edu.sa

**Keywords:** cyclic fatigue resistance, nickel–titanium files, pediatric rotary systems

## Abstract

*Background and Objectives*: Nickel–titanium (Niti) instruments have enhanced root canal cleaning in primary teeth, but file fractures are still common. *Materials and Methods:* This study evaluated the cyclic fatigue resistance of 120 Niti files from four different systems, A: Kedo SG (n = 30); B: Neoendo Pedoflex (n = 30); C: Pedoflex Waldent files (n = 30); and D: Vortex Blue files (n = 30). All the files had similar tip diameters (0.25 mm) and tapers (0.4%) and underwent heat treatment during manufacturing. Cyclic fatigue tests showed notable variations in cycles to fracture (NCF) across groups. All fracture surfaces of the files were assessed through scanning electron microscopy. *Results:* The mean values achieved in the experimental groups (A, B, C) were less than those in the control Group D (976.90 ± 1085.19). Files in Group A demonstrated the highest NCF (697.01 ± 420.09), while Pedoflex files in Group C showed the lowest values (203.88 ± 155.46). Statistical analysis using the Mann–Whitney test revealed significant differences between Group C and Groups A, B, and D and no differences among Groups A, B, and D. *Conclusions:* These findings suggest that Kedo SG and Neoendo Pedoflex files offer comparable cyclic fatigue resistance to Vortex Blue files. In contrast, Pedoflex Waldent files exhibit lower resistance to fracture.

## 1. Introduction

Apical periodontitis is the leading cause of early tooth extractions in primary teeth. Chronic inflammation can disrupt the normal process of root resorption in these teeth, having a significant impact on the growth and eruption of permanent teeth. This can result in conditions such as enamel hypoplasia, abnormal tooth shapes, misalignment, and potentially, odontogenic cysts, which can lead to necrosis of the permanent tooth germ [1]. In vital teeth with signs of reversible pulpitis, pulpotomies are performed to preserve the vitality of the remaining pulp tissue [2]. However, in cases where the pulp is non-vital or irreversibly inflamed, pulpectomy should be considered a primary treatment option to maintain primary teeth until physiological exfoliation, thereby attaining esthetics and functions such as phonetics and mastication [3]. Canal debridement, a critical procedure during pulpectomy, is typically performed using hand files. Nevertheless, the implementation of engine-driven nickel–titanium (Niti) instruments has significantly enhanced the efficacy of canal cleaning and shaping [4]. Its use has also reduced iatrogenic errors, such as canal transportation and ledges [5]. Obturating the canals, which seemed tedious in canals prepared with conventional hand instruments, has also been eased due to the greater taper in most of these files, resulting in better-quality root canal fillings [6]. Studies among pediatric patients have revealed that it positively impacts treatment outcomes, increasing patient acceptance by considerably reducing treatment time [7,8,9]. Despite the noted benefits and wide acceptance of mechanized Niti files among dentists, their influence in treating canals of primary teeth is relatively new [10,11].

Despite the perceived advantages, fatigue-related file separations remain a problem with these files, especially when rotated in curved canals [12]. The reported incidence of instrument fracture varies from 0.7% to 6% [13]. Most Niti file systems fracture abruptly without any prior warning, unlike stainless-steel files, whose flutes unfold, which can be recognized by their shiny surfaces on the file [14,15]. The single-time use of files, as recommended by manufacturers, especially when treating constricted canals, has reduced the incidence of fracture but has not eliminated it [16,17]. A range of methods, including surface treatments and heat treatments, are employed to address the issue. These approaches produce Niti alloys that are more flexible and exhibit enhanced resistance to cyclic fatigue by forming martensite or an R-phase [18,19,20,21].

Additionally, factors such as tip size, file diameter, and the incorporation of novel, cross-sectional, geometrical designs have also been shown to influence the cyclic failure of files [22,23,24]. However, limited details have been available comparing fatigue resistance in heat-treated Niti files with the same tip size [25]. Primary teeth, in comparison to permanent teeth, have short, thin, and curved roots. Using large-taper Niti rotary file systems designed for permanent teeth on these roots poses a risk of lateral perforations. To address this issue, rotary files tailored specifically for primary teeth have been recommended, as they simplify usage in children due to the shorter lengths [6,26]. They are safe to use, resulting in better-quality canal preparations and reduced iatrogenic errors, comparable to those of files designed for permanent teeth [11].

One such file is the Kedo SG gold file (Reeganz Dental Care; Pvt. Ltd., Chennai, Tamil Nadu, India), which is manufactured exclusively for primary teeth. The Kedo rotary files encompass five generations, each characterized by unique heat treatments, surface modifications, and cross-sectional geometries. For this study, the Kedo SG D1 file was utilized, which features a variable taper of 4–8%, a 0.25 mm tip diameter, a triangular cross-sectional design, and a uniform length of 16 mm [26]. Previous studies have demonstrated its efficiency, showing a low incidence of file separations [27]. This allowed for relevant comparisons, as the other files evaluated for cyclic fatigue in this study included those available in 16 mm pediatric lengths with similar cross-sectional designs, 4% taper, heat treatment, and 0.25 mm tip diameters. These include the Neoendo Pedoflex files (Orikam Healthcare, Gurugram, Haryana, India) and Pedoflex files (Waldent Innovations, ‘Pvt. Ltd.’ New Delhi, India).

The sheer magnitude of such files overwhelming the market, questionable standardization, and lack of adequate studies necessitate comparative studies that evaluate their mechanical properties against those of more globally recognized standards [28,29]. In this regard, the Vortex Blue (VB) files (Dentsply Sirona, Ballaigues, Switzerland) served as a control, as prior research indicates that they are better suited for curved canals due to their enhanced flexibility and fatigue resistance, partly due to the titanium oxide coating on the surface of these files [30]. The 4% taper, 0.25 tip size VB file, featuring a convex triangular cross section, quite similar to the experimental files, was utilized. However, due to the VB file’s availability in only adult sizes, a file measuring 21 mm in length was selected.

Given the scarcity of comparative data on pediatric Niti files with similar surface treatments, tip dimensions, and cross-sectional geometries, this study aimed to evaluate the cyclic fatigue performance of three pediatric file systems: Kedo SG, NeoEndo Pedoflex, and Pedoflex files (Waldent), alongside the VB file in simulated curved canal environments. The null hypothesis posited that VB files would demonstrate superior resistance to fracture compared to the other tested files due to their established clinical longevity and the manufacturer’s reputation for producing Niti rotary files.

## 2. Materials and Methods

### 2.1. Ethical Approval

Before commencing the study, ethical approval was obtained from the Standing Committee of Bioethics Research (SCBR) at Prince Sattam bin Abdulaziz University (PSAU), with approval number SCBR-046-2023.

### 2.2. Study Samples

Three systems of Niti rotary files, which are being promoted for pedodontic use due to their similar characteristics, including heat treatment, 16 mm length, triangular cross-section, and a 0.25 mm tip diameter, were subjected to cyclic fatigue testing. VB files with an identical 0.25 mm tip diameter and 4% taper were used as a control group. A sample size calculation was performed using G Power analysis with an effect size of 0.1 and a power of 0.95. All files were visually inspected at 30X magnification for any deformities using a dental operating microscope (Zumax OMS2380, Suzhou, Jiangsu Province, China), and those with noticeable deformities were excluded. A total of 120 standard files were selected and grouped according to the manufacturers: Group A, Kedo SG files (n = 30); Group B, Neoendo pedoflex files (n = 30); Group C, Pedoflex files by Waldent (n = 30); and control Group D, Vortex Blue files (n = 30). Table 1 illustrates a comparison of the files used.

### 2.3. Cyclic Fatigue Resistance Test

**Figure 1:**
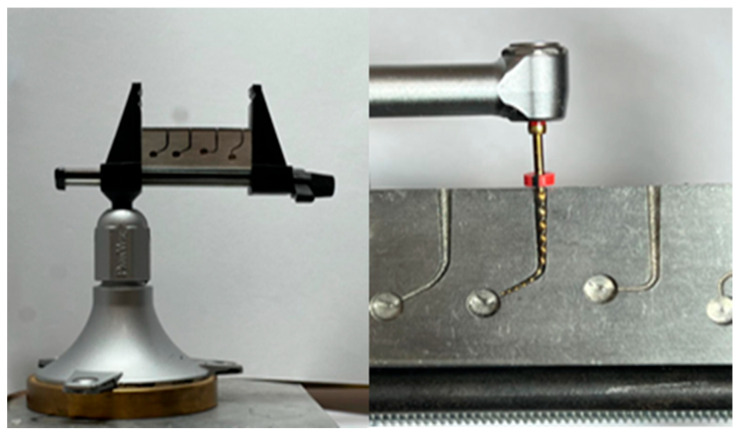
Cyclic fatigue testing apparatus with the test sample.

A specially designed fatigue tester (Denbotix, Bucheon, Republic of Korea) was used to evaluate the resistance of the files to cyclic fatigue. The component consisted of two parts: an artificial metal canal with a 17 mm length, 5 mm radius, and 1.5 mm intracanal diameter, featuring a 60-degree curvature and an arm part that helps firmly hold the handpiece for file rotation, as shown in Figure 1. The study design was derived from a previously validated research framework established by Pruett et al. and from recent studies employing the same methodological approach, as evidenced in the reports by Surme et al. [31,32].

The artificial canal was sealed with a clear acrylic plate secured by screws. This setup allowed for the observation of instrument rotation until fracture and prevented any slippage. The Endo motor handpiece was mounted on a tester that allowed the unhindered placement of each instrument inside the artificial canal. Rotary files were rotated in an endo motor (X-Smart, Dentsply Sirona, Charlotte, NC, USA) using a conventional rotary motion, with the speed and torque recommended by the manufacturer. Before each use of the file for testing, lubricating oil (Millet Franklin, BA, Argentina) was used to reduce friction between the file and the metal canal walls. During each test, the instrument was monitored and visualized through the clear plate until fracture occurred, and the time to fracture was registered in seconds using the digital timer (Timex, Middlebury, CT, USA). The number of cycles to fracture (NCF) was calculated using the equation

NCF = Number of rotations per minute × Time to fracture/60 s

### 2.4. SEM Analysis

The fractured segments were examined using an FEI Quanta 250 FEG scanning electron microscope (Field Electron Ion Company, Hillsboro, OR, USA). Two broken files from each system were analyzed using SEM, and photomicrographs of the fractured regions were captured at various magnifications.

The length of the fractured segment was established using a millimeter ruler (Endo-Eze Ruler, Ultradent Products, Inc., South Jordan, UT, USA).

#### Statistical Analysis

The data were recorded and analyzed using SPSS version 20. Kruskal–Wallis test was performed for group comparisons, and Mann–Whitney U test was used for inter-group comparisons with statistical significance set at *p* ≤ 0.05.

## 3. Results

**Figure 2:**
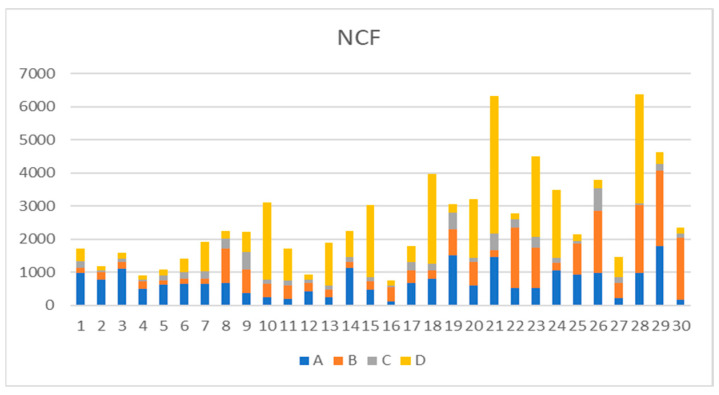
Graphical representation of NCF values of all samples.

The NCF values for samples of all groups are represented as a graph in Figure 2.

As Table 2 shows, the Kolmogorov–Smirnov and Shapiro–Wilk normality tests were performed to measure the central tendency and methods for analysis.

Since the data in Table 2 do not follow a normal distribution, a nonparametric Kruskal–Wallis test was performed to compare different groups. The mean values measured in all groups (A, B, C) were less than those in control group D (976.90). The highest mean NCF values were seen in Group A (697.0191), while the lowest values were in Group C (203.88), as represented in Table 3.

When intergroup comparisons were made using the Mann–Whitney test, Groups A and B significantly differed from Group C, with *p*-values < 0.001. However, no such difference was observed between the control Group D and Groups A and B, as seen in Table 4.

**Figure 3:**
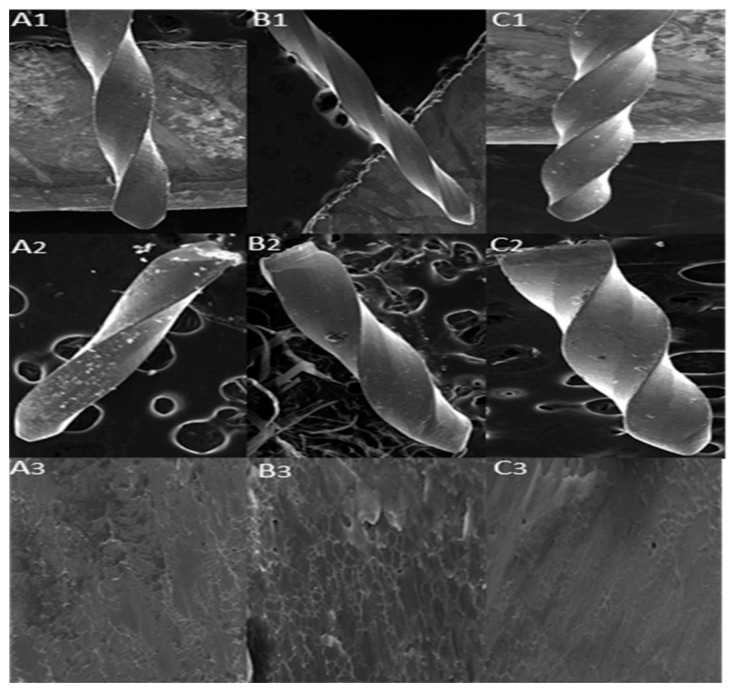
SEM Photomicrographs. (**A1**) 25 04% tapered Kedo SG file, (**A2**) fractured segment of the Kedo SG file, (**A3**) fracture zone at 1500× magnification, (**B1**) 25 04% tapered Neoendo Pedoflex file, (**B2**) fractured segment of the Neoendo pedoflex file, (**B3**) fracture zone at 1500× magnification, (**C1**) 25 04% tapered Waldent Pedoflex file, (**C2**) fractured segment of the Waldent Pedoflex file, (**C3**) fracture zone at 1500× magnification.

The fractured segment was visualized, and an SEM analysis was performed. The photomicrographs demonstrated striation patterns and fracture areas, which are typical indicators of cyclic fatigue failure, as seen in the fracture plane images of the files. Shown in Figure 3 are representative SEM photomicrographs at 50× magnification of the tested tools and their fractured sections. The fractured surface of the fragment was examined at 1500× magnification, revealing the striations and zones of overload.

**Figure 4:**
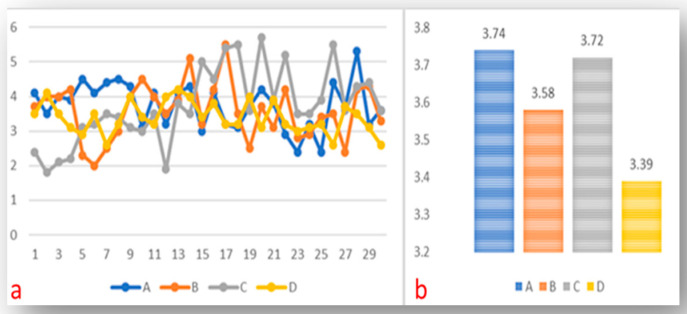
(**a**) Graphical representation of the length of fractured segments of files; (**b**) mean values of fractured segments.

The mean lengths of fractured segments measured in mm are displayed in Figure 4.

## 4. Discussion

The file’s tip size, diameter, and cross-sectional design could also influence these results. To standardize the study, the authors utilized files with similar surface treatments, cross-sectional designs, and tip sizes: the Kedo SG, Neoendo Pedoflex, and Pedoflex files from Waldent, and compared them to a commonly used, heat-treated Niti system, the VB. However, the findings revealed that both the Kedo SG and Pedoflex files exhibited comparable performance to the VB file. Notably, only the Pedoflex file from Waldent showed statistically inferior resistance to fracture. Thus, the null hypothesis was partly rejected, as the cyclic fatigue resistance difference was insignificant in two of the three experimental files

While Niti files are continuously being manufactured and supplied due to the growing demands of dentists, there is an increasing need to select reliable file systems [33]. Moreover, Niti instruments do not have any standard guidelines for manufacturers to comply with [34]. The practitioners now have a plethora of instruments to choose from. This choice of files is often influenced by clinicians’ trust in manufacturers, based on their clinical experience and peer recommendations. As the use of Niti files in pediatric patients was first reported relatively recently, studies on their use, properties, and clinical performance are limited [10]. Many practitioners continue to use the Niti file systems similar to those used in adult patients [27,35]. However, a need for files of shorter length arose in pediatric patients due to their limited mouth opening and the comparatively shorter root lengths in these teeth [36]. More recently, dentists have reported an increase in the acceptance and use of rotary instrumentation in primary teeth, owing to the facilitation of homogenous fill, reduced debris extrusion, and shortened working time [37,38]. Its use could benefit pediatric patients, especially in quadrant dentistry and in patients under general anesthesia. Results from India suggest that around 50% of pediatric dentists prefer using rotary instrumentation to shape canals in primary teeth [6]. However, a cross-sectional study in Saudi Arabia showed that only 21.5% of the practitioners used rotary instruments [39]. In both the studies mentioned above, files from pro-taper systems were most commonly used, followed by dedicated pedodontic files, such as Kedo S and Prime pedo files, in Saudi Arabia [6,39].

Nevertheless, file separation while using Niti files continues to be an impediment and can negatively impact the prognosis [40]. While either torsional or cyclic fatigue can lead to file separations, cyclic fatigue is more commonly the culprit, which can directly influence the prognosis of treatment [41]. Repeated bending of files in curved canals could lead to cyclic fatigue. The more the taper and size of the file, the lower its resistance to cyclic fatigue. Although some of these factors may be operator-dependent, technological advances and the incorporation of heat and surface treatment have increased the file’s resistance to fatigue [42,43].

This study tested the rotary files in artificial metal canals designed on a stainless-steel block with known diameters, depths, and curvatures, as seen in previous studies investigating cyclic fatigue [44]. In comparison, others have utilized different methodologies, including bending instruments against three points and using inclined planes [45]. The authors in this study used an oil lubricant to reduce friction and heat generation during movement in a metal canal [46]. Cyclic fatigue is usually evaluated in either static or dynamic motion. Dynamic back-and-forth movement has been touted as a means to replicate the clinically employed pecking motion. It could improve the cyclic fatigue of Niti files due to a broader distribution of stresses [47,48]. Nevertheless, studies have also shown that it may not accurately replicate clinical situations as the number of oscillations is far less than in the dynamic fatigue testing models [49]. On the other hand, during static motion, there is a higher chance of stress concentration at the point of maximum curvature due to the lack of axial movement of files within the canals [50]. In this study, the cyclic fatigue test was conducted in static motion due to the ease of standardization and consideration of the difficulties in accurately setting up a dynamic model.

The superior cyclic fatigue resistance of VB files compared to other files is attributed to their controlled memory and blue heat treatment [30,51,52]. The NCF values in some research on VB files varied due to the use of different tapers and tip sizes compared to this study [51,53]. Differences could also be due to stress concentration in the static model used in our research and the variation of canal curvatures [25]. Time to fracture and NCF value variations between studies could also be due to the fact that guidelines for production and testing are still lacking, and none of the previous work in the field seems to suggest it [54].

Sufficient studies regarding cyclic fatigue in pediatric Niti files are lacking. Nevertheless, a study conducted in Turkey compared two groups of heat-treated pediatric Niti files. The T-endo Must files (TEM; Dentac, Istanbul, Türkiye), manufactured for use in reciprocation motion, exhibited better cyclic fatigue resistance compared to the AF baby file (ABF; Fanta Dental Materials Co., Shanghai, China), which was used in rotation. Although both file systems were heat-treated, the difference in that study, as noted by the authors, could be attributed to the type of instrument motion [55]. In contrast, a study on two 0.4 tapered pediatric file systems found that the AF baby rotary files showed better resistance to cyclic fatigue than the i3 Gold deciduous teeth rotary files, with a mean NCF value (1516 ± 204.05) higher than that observed in this study [44]. Recently, significantly higher mean NCF values were observed when four pediatric rotary files were used, with mean values ranging from 453.65 to 2668.10. Variations in metallurgy, file geometry, and study design may also contribute to these variations [32]. Another clinical prospective study in India examining the incidence of fracture in Kedo S pediatric Niti files suggests that these files have a low incidence of separation when used according to the manufacturer’s guidelines. When file separations occurred, it was usually in the apical third [27].

Recent investigations have shown that variations in cross-sectional design, material alloy, and heat treatment have a significant impact on cyclic fatigue resistance. Specifically, a comparative evaluation of ten endodontic files featuring diverse cross-sectional geometries—namely, double S, variable, triangular, S-shaped, and rectangular—revealed that the S-shaped design exhibits markedly superior cyclic fatigue performance [56]. This finding corroborates earlier research by Kaval et al., which indicated that files with S-shaped geometry outperformed others in terms of fracture resistance [57]. Further supporting evidence comes from a study examining files with distinct cross-sectional shapes (parallelogram, triangular, and S-shaped) used in clinical settings, where the S-shaped files demonstrated the highest resistance to fracture [58]. Another investigation focused on files subjected to similar heat treatment but presenting different cross-sectional designs (S versus triangular). The S-shaped files, characterized by two cutting blades, exhibited enhanced cyclic fatigue resistance due to reduced surface contact, minimized stress concentration, and a lower mass percentage when compared to their triangular counterparts, which featured three cutting blades [59]. In a controlled setting with a constant taper of 0.4%, the performance of various cross-sectional geometries was reassessed, reaffirming the S-shaped design as superior, as reported in the study conducted by Ersoy et al. [60]. The reasons behind the suboptimal performance of certain cross-sectional designs remain somewhat enigmatic. Some researchers propose that the core material’s properties, particularly at points of maximal stress, are critical determinants of CFR, while others dispute any significant effect. Additional research indicates that reducing the metal mass at locations of peak stress can enhance the CFR of nickel–titanium rotary files [32,61,62]. Moreover, the manufacturing process itself has been identified as a contributory factor affecting CFR, highlighting the need for a comprehensive understanding and strategic design in file development [63].

As is commonly observed, most instruments fracture in the apical third of the root canals, typically in the area of maximum curvature [64]. Terauchi et al. suggested that retrieval is adversely affected if the length of the fractured instrument exceeds 4.5 mm [65]. In this study, the fractured segments from all files were below 4 mm, with no significant difference. The data indicated that the VB file system exhibited the lowest mean values. Consistent with our findings, recent reports indicated that the lengths of fractured segments ranged between 3.43 mm and 3.65 mm [32]. In contrast, a recent study identified mean fracture lengths ranging from 7.25 mm to 9.12 mm [44]. Variations in performance may stem from the use of different file systems and the taper and wire technologies employed during the manufacturing process.

The methodology used in this study to test for cyclic fatigue was similar to that in many previous studies, where artificial metal canals of known curvatures were manufactured [44]. However, the static testing model and its dependence on simulated metal canals may not completely replicate the clinical dynamics, as factors such as canal anatomy variability and operator technique influence outcomes. Therefore, the static movement of files in the canals should be considered a limitation in this study and, as previously highlighted, this approach may have led to stress concentrations and could precipitate early fatigue. However, because passive movement was consistently applied across all experimental groups, the resulting findings should still offer a robust comparative analysis. Additionally, the rigorous file selection methodology, which utilized standardized artificial canals and controlled testing conditions, ensured internal validity by isolating variables such as taper, tip size, and cross-sectional design. Furthermore, the study did not compare files with different cross-sectional designs and tip sizes, which could have provided a more comprehensive comparison and a better understanding of selecting files for clinical use. Future studies should aim to address the limitations of current research by using dynamic testing models. Additionally, incorporating natural teeth and clinical trials would provide better insights into the performance of Niti files with greater clinical relevance. Future studies should also evaluate the actual number of rotations required to prepare canals in natural teeth, considering their size, number, and degree of curvature. This information can be used to correlate with the NCF values achieved with Niti files, thereby determining the approximate number of uses of these files before disposal.

## 5. Conclusions

Within the limitations of this study, Kedo SG and Neoendo Pedoflex demonstrated comparable fatigue resistance to VB, indicating their potential suitability for clinical applications. In contrast, Waldent Pedoflex displayed lower fatigue resistance, which may necessitate careful consideration for clinical use to ensure optimal patient outcomes. Although the NCF values for this file were lower, its clinical application may still be viable, particularly for single use as recommended by most manufacturers.

## Figures and Tables

**Table 1:** Description of the samples used in the study.

Variable	Control File	Experimental File		
File	Vortex Blue	Kedo SG (D1)	Neoendo Pedoflex	Pedoflex
Manufacturer	Dentsply Sirona, Ballaigues, Switzerland	Reeganz Dental Care Pvt. Ltd., Chennai, TN, India.	Orikam Healthcare, Gurugram, Haryana, India.	Waldent Innovations, Pvt.Ltd., New Delhi, India.
Tip size	0.25	0.25	0.25	0.25
Taper	4%	4% (variable 4–8%)	4%	4%
Cross-sectional design	Convex triangular	Triangular	Triangular	Triangular
Surface treatment	Heat treated/titanium oxide	Heat-treated	Heat-treated	Heat-treated
Tip	Non-cutting	Non-cutting	Non-cutting	Non-cutting

**Table 2:** Test of normality.

		Kolmogorov–Smirnov			Shapiro–Wilk		
	Group	Statistic	df	Sig	Statistic	df	Sig
	A	0.122	30	0.200 *	0.939	30	0.077
**NCF**	B	0.264	30	0.000	0.757	30	0.000
	C	0.207	30	0.002	0.808	30	0.000
	D	0.246	30	0.000	0.775	30	0.000

* Nonsignificant difference with *p*-value ≤ 0.05; Test results show non-parametric distribution.

**Table 3:** Non-parametric test based on mean NCF values.

Group	n	Mean with Std. Dev	Median	Inter Quartile Range
A	30	697.01 ± 420.09	645.33	600.15
B	30	661.76 ± 655.09	386.47	721.28
C	30	203.88 ± 155.46	161.33	116.38
D	30	976.90 ± 1085.19	414.19	576.25

**Table 4:** Intergroup comparison.

Group	Groups	Mean Difference	Z	*p*
A	B	35.25	1.373	0.17
	C	493.13	5.392	<0.001 ***
	D	−279.88	0.592	0.554
B	C	457.87	4.210	<0.001 ***
	D	−315.14	0.627	0.531
C	D	−773.02	4.259	<0.001 ***

Z = Mann–Whitney U test for inter-comparison; *** very highly significant.

## Data Availability

Available upon suitable request.
